# Breeding zebra finches prioritize reproductive bout over self-maintenance under food restriction

**DOI:** 10.1242/bio.060417

**Published:** 2024-11-05

**Authors:** Victoria M. Coutts, Kevin Pham, Gabriella Gilbert, Haruka Wada

**Affiliations:** Department of Biological Sciences, Auburn University, Auburn, AL 36849, USA

**Keywords:** Nutritional stress, Stress, Corticosterone, Glucose, Songbirds, Reproduction

## Abstract

Reproduction requires high amounts of energy, and challenging environments during breeding can force parents to prioritize their current reproductive bout over self-maintenance or vice versa. However, little is known about how common stressors, such as food restriction, can influence these trade-offs during breeding, and the physiological mechanisms for these trade-off decisions. In this study, adult zebra finches (*Taeniopygia castanotis*) were subjected to a control diet (*ad libitum*) or a 40% food restriction while raising nestlings and fledglings, and we measured body mass, furculum fat, plasma corticosterone (CORT) and blood glucose levels of the parents at the time of pairing, when their offspring fledged, and when their offspring reached nutritional independence. We also measured body mass and growth rate in the offspring from hatching until the end of the treatment period. Food-restricted parents had lower body mass when their offspring fledged and reached nutritional independence and higher baseline CORT when their offspring fledged compared to controls. Offspring did not differ in body mass or growth rate between treatment groups. However, there was no effect of food restriction on parents’ furculum fat, baseline glucose, the adrenocortical response, or the glucose response. Furthermore, path analysis results suggest that alterations in baseline glucose is the primary driver of changes in body mass in parents and offspring brood mass. Taken together, these results suggest that food restriction during chick rearing in a short-lived passerine drives parents to prioritize their current reproductive bout over self-maintenance, and glucose could potentially be a mechanism for diverting energy toward parental effort.

## Teaser

**Summary:** When faced with food restriction, short-lived zebra finch parents prioritized current reproduction over maintaining their own body mass, possibly through altering glucose levels.

## INTRODUCTION

In an environment with finite resources, organisms balance life-history trade-offs between reproduction and self-maintenance, number and size of offspring, or body size and age at first reproduction (Stearns, 1989). Decisions on how resources are allocated to one life-history trait over another, particularly under periods of high energetic demand, such as breeding, can greatly impact fitness outcomes. During such periods, an unfavorable environment and other stressors can negatively impact an organism's body condition and their chances for future reproduction (Stearns, 1992; Roff, 2002), amplifying the cost of reproduction. For individuals with low residual reproductive value, resource allocation towards current reproduction should be higher than allocation towards self-maintenance, while individuals with high residual reproductive value should allocate more resources towards self-maintenance than current reproduction (Williams, 1966; Stearns, 1992). Under van Noordwijk and de Jong's (1986) model, a trade-off between reproduction and survival (e.g. self-maintenance) precipitates when resource availability is low, and allocation varies. Regardless of residual reproductive value, once parents invest a certain amount of energy into the current reproductive bout, the cost to abandon the reproductive attempt increases, and parents are more likely to keep raising the young. In such a scenario, individuals with little access to resources face a greater degree of trade-off, altering the magnitude of allocation towards reproduction.

Food shortage is one of the most common stressors in the wild that can increase the cost of reproduction. Thus, the reproductive trade-offs between self-maintenance and current reproduction become more consequential. Short-lived species in particular could be vulnerable to food shortage during reproduction as they tend to have lower residual reproductive value compared to long-lived species (Williams, 1966; Stearns, 1992). Consequently, they may face different trade-off outcomes when balancing between life-history traits under conditions with limited resources compared to long-lived species. Zebra finches (*Taeniopygia castanotis*) are particularly unique short-lived passerines due to their atypical life-history. Although most short-lived passerines are seasonal breeders and rarely breed more than once or twice a year, zebra finches live in arid regions where resources are scarce (Morton et al., 2011) and are aseasonal, opportunistic breeders, preferring to breed when resources become available (Zann et al., 1995; Zann, 1996). Depending on resource availability, these zebra finches may have more chances to breed than seasonal breeders each year (Zann et al., 1995), which would increase their residual reproductive value. Thus, for zebra finches, parental decisions on reproductive trade-offs in response to food shortage may not follow the same patterns expected of a typical short-lived species (i.e. more investment into reproduction due to lower residual reproductive value). In particular, these decisions become apparent if food availability decreases after reproduction has already begun. For example, zebra finch parents exposed to lower quality food (i.e. low macronutrient content) spent less time brooding their offspring, showing a prioritization toward self-maintenance (Krause et al., 2017). However, we still do not completely understand the limitations of poor nutritional conditions on reproductive decisions in zebra finches or how they make investment decisions while breeding (Griffith et al., 2021).

Since direct manipulation of food availability is difficult in the wild, researchers mimic food shortage in controlled laboratory environments. To add ecological relevance, studies tend to add other factors, such as mixing inedible items in food to mimic foraging effort or increasing brood size (Lemon, 1993; Kriengwatana et al., 2013, 2015). While these studies, particularly the increased foraging studies, can more effectively reflect wild conditions, they often answer different questions than studies that simply restrict food. For instance, birds can compensate for brood size manipulation or artificially increased foraging effort by simply collecting more food. On the other hand, a direct manipulation of food quantity can reflect more on actual lack of food availability, as individuals do not have the ability to gain further resources, and therefore can answer questions about how food shortage itself influences parental care in zebra finches. Furthermore, the physiological mechanisms of energy prioritization during breeding under food shortage in zebra finches remain to be tested.

Glucocorticoids (i.e. main glucocorticoids being corticosterone or CORT in birds) are metabolic hormones released by the hypothalamic-pituitary-adrenal (HPA) axis that mediate changes in resource allocation in response to environmental changes (Wingfield, 1994; Wingfield et al., 1998) and are thought to be linked to environmental condition and resource allocation during breeding. Moreover, the baseline and magnitude of the adrenocortical responses have been hypothesized to mediate life-history transitions across vertebrate taxa (Wada, 2008). Baseline levels, or the levels of CORT that are normally circulated throughout the body, can regulate multiple physiological and behavioral systems, and promote homeostasis (Sapolsky et al., 2000; Romero et al., 2009). Furthermore, in zebra finches, poor nutrition is known to increase baseline CORT (Honarmand et al., 2010; Lynn et al., 2010). Baseline CORT may also play a role in sustaining and/or maintaining breeding attempts/effort, as baseline CORT levels positively correlate with brood value in passerines (Wingfield and Sapolsky, 2003; Bokony et al., 2009). Therefore, when breeding parents experience a sudden drop in food availability, baseline glucocorticoid levels are likely to be altered and influence whether self-maintenance or current reproductive bout will be prioritized. Changes in food availability can also affect individual's ability to mount an adrenocortical response. Indeed, the magnitude of the adrenocortical response can drastically change depending on life history stage, prior stress history, and when under chronic stress (Sapolsky et al., 2000; Romero et al., 2009; Foley and Kirschbaum, 2010). For example, the adrenocortical response is attenuated in king penguins during the chick brooding stage compared to earlier breeding stages (Viblanc et al., 2016), presumably to prevent overactivity of the adrenocortical response and prioritize reproduction, highlighting the importance of modifying stress responses during life history stages/transitions in some avian species (i.e. incubating versus brooding, nonbreeding versus breeding; Love et al., 2004; Bonier et al., 2009). If baseline glucocorticoids and the adrenocortical response play such essential roles in life-history transitions, it is possible that these physiological measures may mediate parental response to food restriction in the middle of breeding in zebra finches.

Although glucocorticoids have been proposed to influence life-history decisions, other physiological processes such as glucose may play a key role in facilitating such trade-offs. Baseline glucose is correlated with life-history traits such as body mass and reproductive investment, with higher glucose levels increasing with higher demands of growth and reproduction (Tomasek et al., 2018). This is because glucose is thought to be the primary source of energy for processes that require immediate high levels of energy, including reproduction (Braun and Sweazea, 2008; Kalinski et al., 2015; Scanes and Braun, 2013) rather than other macromolecules such as fats. Therefore, glucose regulation in response to environmental stressors may be another important component of the stress response to maximize function, survival or fitness. This may be particularly important in response to food restriction, as this could lead to insufficient glucose levels that reduce the amount of energy available for reproduction. Indeed, increased foraging costs can lead to increased levels of baseline glucose in adult zebra finches (Montoya et al., 2018). The glucose response (i.e. the difference between stimulated and baseline glucose levels) to an acute restraint can follow the same pattern as glucocorticoids and increase over time, but the response varies depending on time of day and breeding stage in seasonal breeding birds (Remage-Healey and Romero, 2000; Deviche et al., 2016). However, the glucose response has not yet been tested under food restriction during breeding. Although some studies have investigated the effects of stressors on the glucose response, this is not always related to glucocorticoids, calling to question our knowledge on the glucose stress response and how they vary across life history stages (Neuman-Lee et al., 2020; Taff et al., 2022). Additionally, applying food restriction and measuring its effects on physiological mechanisms such as glucocorticoids and glucose in breeding parents is also a topic not well covered by literature. Thus, we still know little about how food restriction may influence baseline glucose or the glucose stress response in opportunistic breeders such as zebra finch parents and how it may act independently of the adrenocortical response.

To fill these knowledge gaps, we exposed breeding parents to food restriction and measured their body mass, fat scores, plasma glucocorticoid concentrations, and blood glucose levels from pairing until offspring are nutritionally independent, in captive colony of zebra finches (*Taeniopygia castanotis*). We applied food restriction after parents had begun the breeding process and invested energy into reproduction. Previous work has shown that parents are more willing to tolerate inadequate energy intake when the chances of a successful brood at the time of energy restriction is higher than other breeding stages (Groscolas et al., 2008). Here, we tested two hypotheses: (1) parents would prioritize resource allocation toward their reproductive bout over self-maintenance, and (2) the mechanism for this resource allocation is through changes in the glucocorticoids and/or glucose levels. If parents prioritize resources toward their reproductive bout, we predict lower body mass and furculum fat in the food-restricted parents and that body mass would decrease as parents invest more into reproduction, i.e. as the mass of their brood increases. If parents prioritize reproduction, we would also not expect differences in body mass or growth rate in food-restricted offspring compared to controls. Additionally, we predicted that higher levels of baseline glucocorticoids and an attenuated adrenocortical response in food-restricted parents, typical patterns shown under chronic stress. If glucocorticoids are related to glucose levels in zebra finches, and higher glucose levels correlate with higher demands of reproduction (Tomasek et al., 2018), we would expect the same patterns in baseline glucose and the glucose response as observed in baseline glucocorticoids and the adrenocortical response with the increased energetic and reproductive demand in food-restricted parents. If glucocorticoids and/or glucose levels are mechanisms for potentially observed resource allocation, we predict baseline glucocorticoids, baseline glucose levels, changes in baseline glucocorticoids, and/or changes in baseline glucose levels are associated with changes in parental body mass and offspring brood mass at demanding times of breeding such as fledging. Since glucocorticoids and glucose may not always be related (Taff et al., 2022), we used path analyses to determine factors that drive changes in parent body mass.

## RESULTS

### Body mass

In parents, food restriction altered body mass, and there was a significant timepoint–treatment interaction (*F*_2,86_=3.88; *P*=0.024). Prior to the start of the treatment (at time of pairing), parents in both groups had a similar body mass (t_86_=−1.58; *P*=0.613; Table 2, Table 3, Fig. 2A). However, parents’ body masses declined with food restriction. As expected, food-restricted parents had lower body masses than control parents when the offspring fledged (t_86_=−4.28; *P*=0.001; Table 3, Fig. 2A) and when the offspring reached nutritional independence (t_86_=−3.13; *P*=0.028; Table 3, Fig. 2A). Specifically, at fledging, parents in the food-restricted group lost 7.01% of body mass on average compared to their mass at pairing, while control parents only lost 1.50%. We also detected a sex effect, in which males had significantly lower body mass than females (*F*_1,39_=4.27; *P*=0.046). However, in offspring, despite a significant timepoint–treatment interaction (*F*_6,493_=2.67; *P*=0.015), food restriction did not impact body mass at any timepoint (t_21_≥−3.63; *P*≥0.065; Fig. 3A). There was also no effect of food restriction on growth rate in offspring (*F*_1,21_=3.55; *P*=0.074; Fig. 3B).

**Fig. 1.**
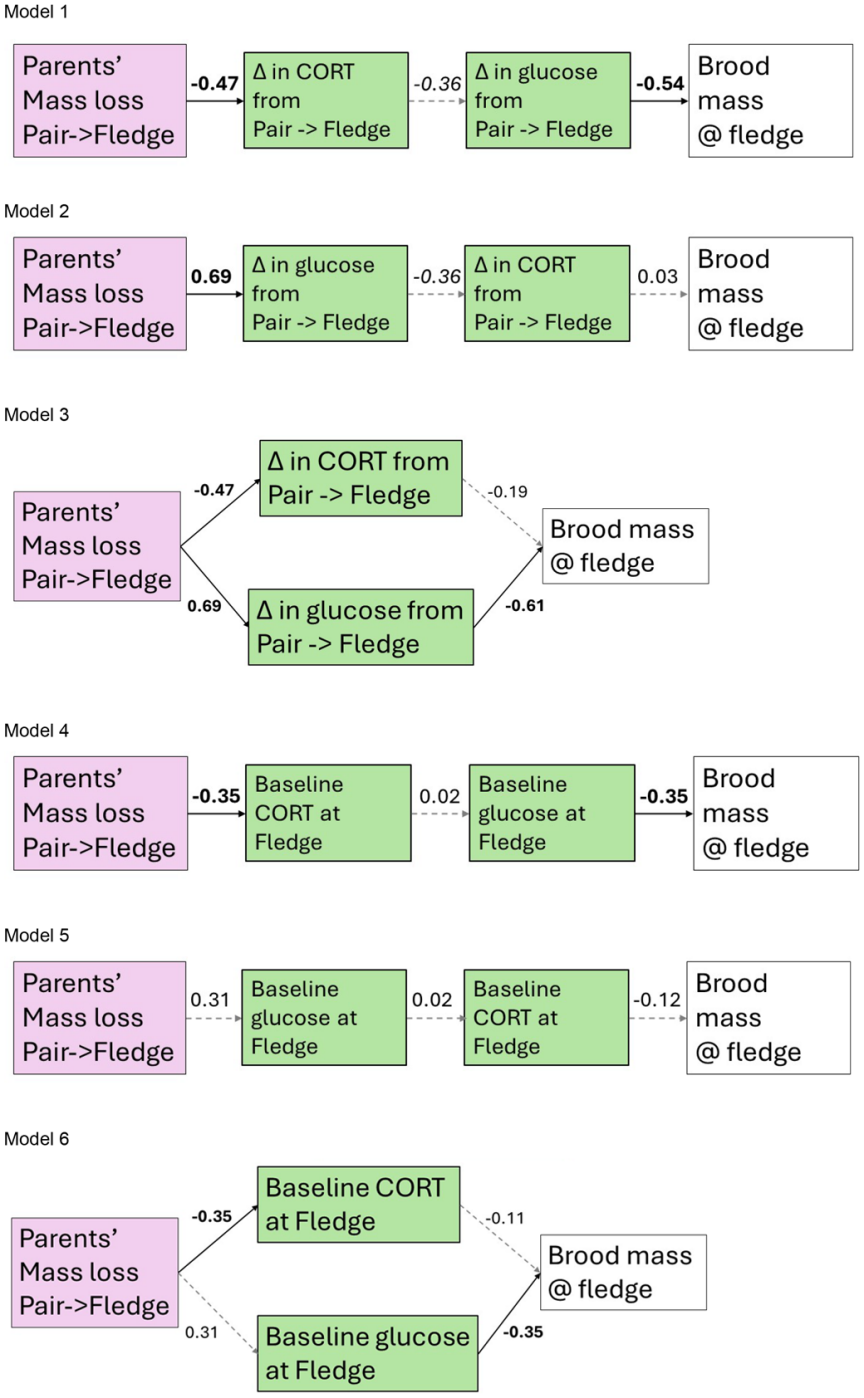
**Standardized path coefficients for models in Tables S1 and S2.** Significant paths are shown with solid lines with coefficients in bold. Non-significant paths are shown with dashed lines.

**Fig. 2.**
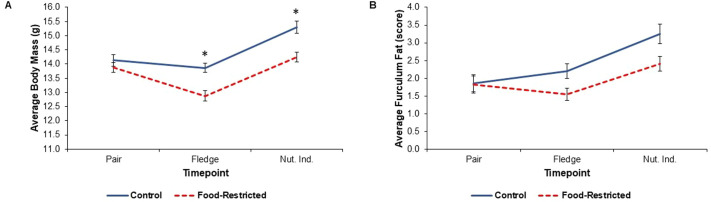
**Body mass and furculum fat score of food-restricted and control parents.** Body mass and furculum fat were collected from 11 males and 12 females in food-restricted nests and 11 males and 11 females in control nests at each timepoint. (A) Body mass results from a linear mixed model are plotted at three timepoints; at pairing (treatment: t_86_=−1.58; *P*=0.613), at fledging (treatment: t_86_=−4.28; *P*=0.001), and nutritional independence (Nut. Ind.; treatment: t_86_=−3.13; *P*=0.028). Raw means±s.e.m. are plotted. Asterisk denotes a statistically significant difference between treatment groups at a particular timepoint based on post-hoc tests for each timepoint. (B) Furculum fat results from a cumulative link mixed model shows there is no significant effect of food restriction on furculum fat in parents (z=0.09; *P*=0.929). This experiment has not been replicated.

**Fig. 3.**
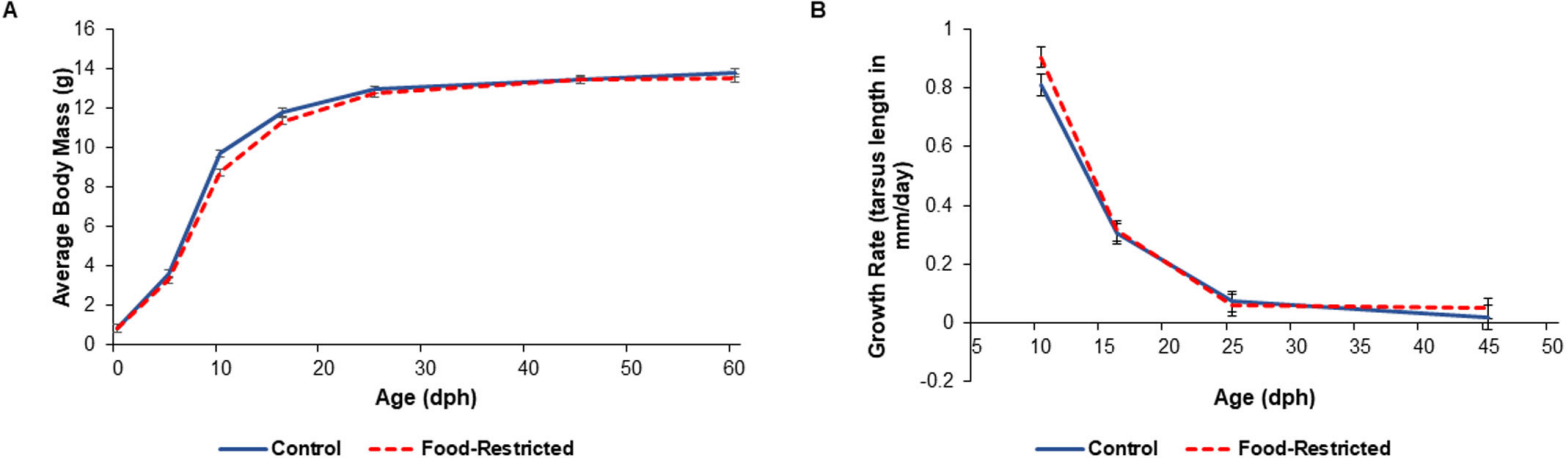
**Body mass and growth rate of food-restricted and control offspring.** Body mass and growth rate was collected from the offspring of 12 food-restricted nests and 11 control nests at each timepoint. These results from linear mixed models show that food-restricted offspring do not differ from controls in (A) body mass (treatment at each timepoint: t_21_≥−3.63; *P*≥0.065), or (B) growth rate (treatment: *F*_1,21_=3.55; *P*=0.074). Raw means±s.e.m. are plotted. Offspring were measured for body mass at 0, 5, 10, 16, 25, 45, and 60 dph and tarsus length at 5, 10, 16, 25, and 45 dph. This experiment has not been replicated.

**Table 1.**
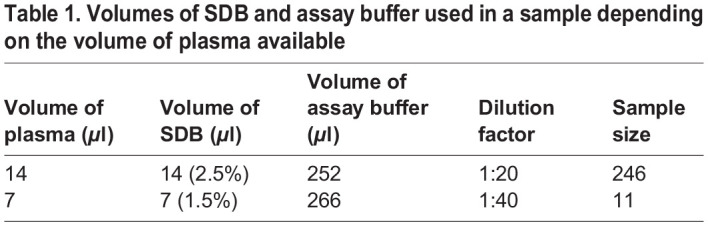
Volumes of SDB and assay buffer used in a sample depending on the volume of plasma available

**Table 2.**
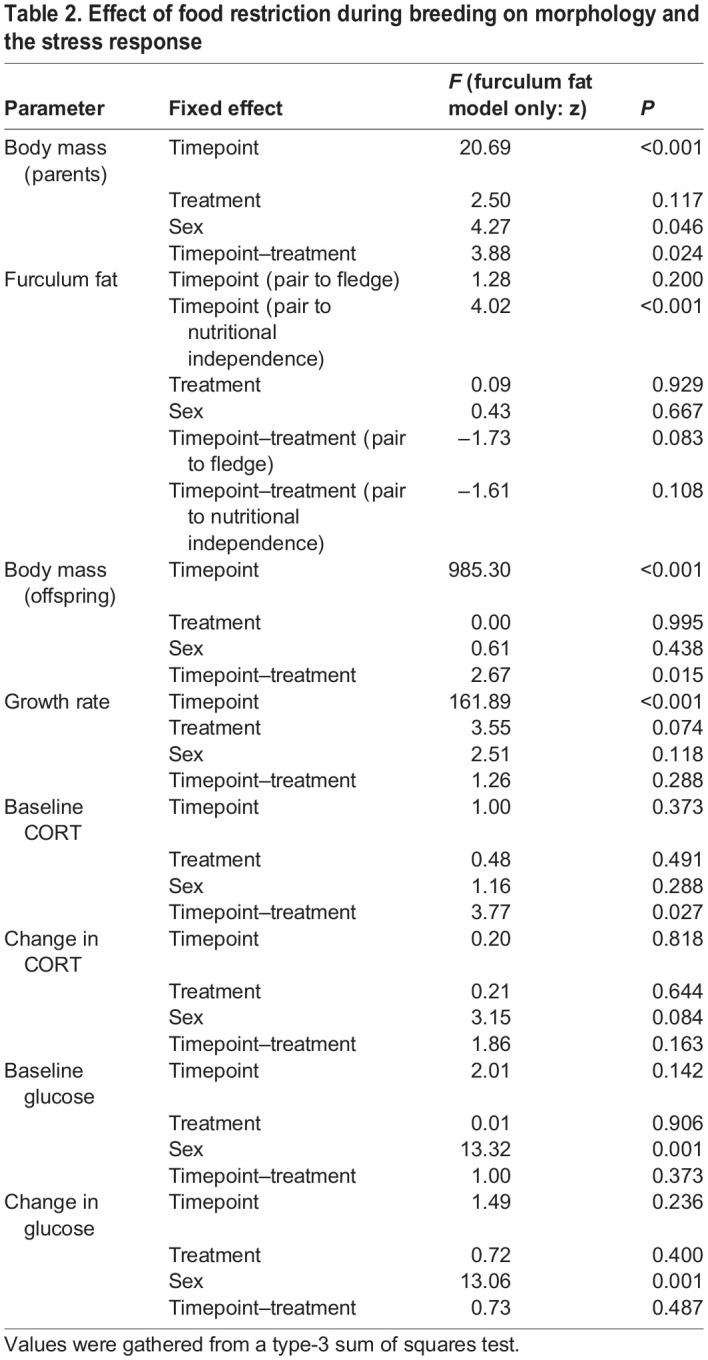
Effect of food restriction during breeding on morphology and the stress response

**Table 3.**
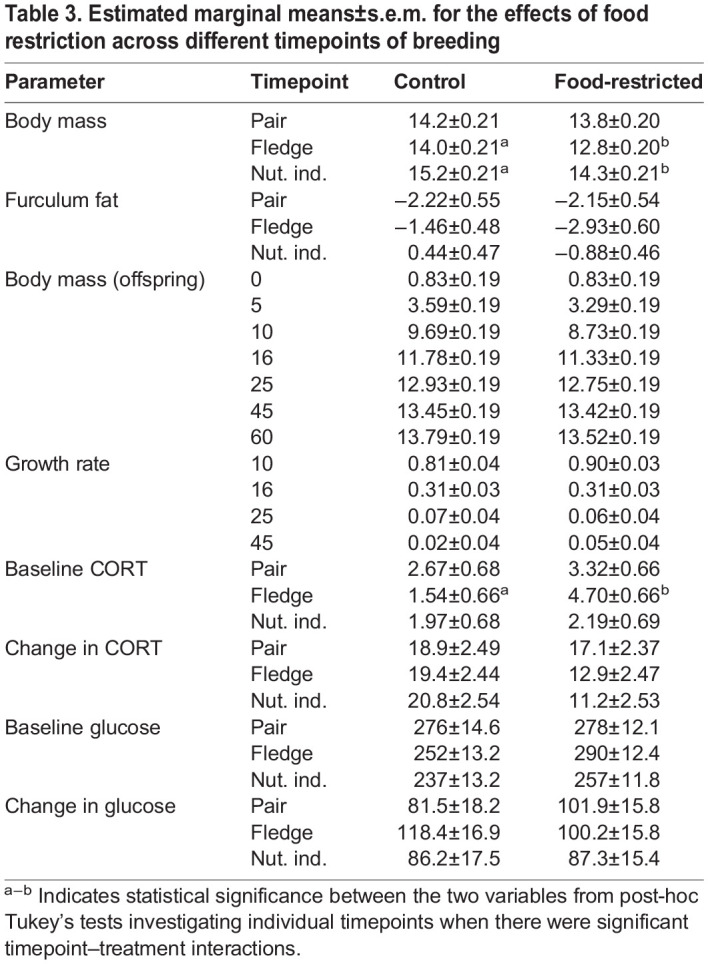
Estimated marginal means±s.e.m. for the effects of food restriction across different timepoints of breeding

### Furculum fat

Overall, there was no main effect of food restriction on furculum fat scores compared to controls (z=0.09; *P*=0.929; Table 2, Fig. 2B). There were no significant interactions between timepoint and treatment (Table 2, Fig. 2B).

### Corticosterone

**Fig. 4.**
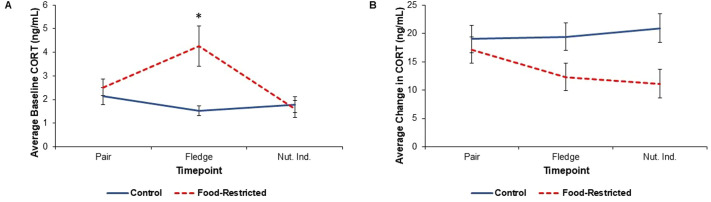
**CORT results in food-restricted (*n*=12) and control (*n*=11) parents.** Blood samples to measure CORT were collected from 11 males and 12 females in food-restricted nests and 11 males and 11 females in control nests at each timepoint. (A) Results from a linear mixed model investigating the effect of food restriction on baseline corticosterone (CORT) (treatment: t_79_=0.69, *P*=0.982 at pairing; t_79_=3.41, *P*=0.013 at fledging; t_79_=0.23; *P*=1.000 at nutritional independence). Raw means±s.e.m. are plotted. Asterisk denotes a statistically significant difference between treatment groups at a particular timepoint based on Tukey's post-hoc tests for each timepoint. (B) Linear mixed model shows food restriction does not impact the adrenocortical response (treatment: *F*_1,76_=0.21; *P*=0.644). Raw means±s.e.m. are plotted. This experiment has not been replicated.

Food restriction altered baseline CORT in breeding birds that was shown by the significant treatment–timepoint interaction (treatment­–timepoint interaction: *F*_2,79_=3.77; *P*=0.027; Table 2). Prior to the start of treatment, there was no difference between treatment groups in baseline CORT (treatment at pairing: t_79_=0.69; *P*=0.982; Table 3, Fig. 4A). However, at fledging [i.e. when the youngest nestling reached 18 days post hatch (dph)], food-restricted parents had significantly higher baseline CORT than the control parents (t_79_=3.41; *P*=0.013; Table 3, Fig. 4A). This effect was transient and disappeared when offspring reached nutritional independence (i.e. when the oldest nestling reached 44 dph) despite the fact that birds were still undergoing food restriction (t_79_=0.23; *P*=1.000; Table 3, Fig. 4A). There was also no main effect of sex on baseline CORT (*F*_1,39_=1.16; *P*=0.288). Overall, food restriction did not impact the adrenocortical response (treatment: *F*_1,76_=0.21; *P*=0.644; Fig. 4B, Table 2).

### Glucose

**Fig. 5.**
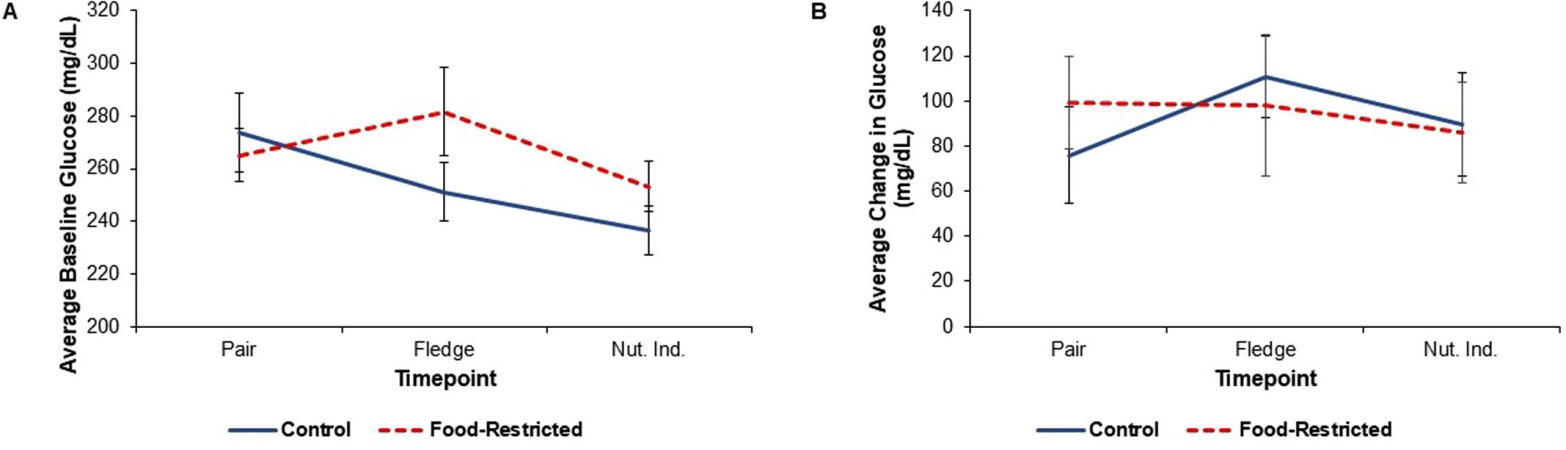
**Glucose results in food-restricted (*n*=12) versus control (*n*=11) parents.** Blood samples to measure glucose were collected from 11 males and 12 females in food-restricted nests and 11 males and 11 females in control nests at each timepoint. Parents were measured at three timepoints; at pairing, at fledging, and nutritional independence (Nut. Ind.). (A) Linear mixed model shows food restriction did not impact baseline glucose (treatment: *F*_1,60_=0.01; *P*=0.906). Raw means±s.e.m. are plotted. (B) Linear mixed model shows there is no effect of food restriction on the glucose response (treatment: *F*_1,52_=0.72; *P*=0.400). Raw means±s.e.m. are plotted. This experiment has not been replicated.

There was no significant effect of food restriction on baseline glucose (treatment: *F*_1,60_=0.01; *P*=0.906; Fig. 5A). We detected a significant sex effect where males had higher baseline glucose than females regardless of treatment (sex: *F*_1,35_=13.32; *P*<0.001). There was no significant interaction between treatment and timepoint or treatment and sex. Treatment did not impact the glucose response (*F*_1,52_=0.72; *P*=0.400; Table 2, Fig. 5B). In general, males had a stronger glucose response than females regardless of treatment (sex: *F*_1,35_=13.06; *P*<0.001). Similar to the baseline glucose results, there was no significant interaction between timepoint and treatment.

### Path analysis

Path analyses revealed that parents’ mass loss between pairing and when offspring fledged affected both change in CORT and glucose between pairing and fledging. However, brood mass at fledging is mediated by glucose, rather than CORT. The brood mass at 16 dph was explained the most by model 3 in which the model explained 33.3% of variation in brood mass at 16 dph (Table S1). In contrast, parents’ mass loss only affected baseline CORT when young fledged and did not affect baseline glucose at fledge (models 4 to 6; Fig. 1). On the other hand, models connecting baseline glucose at fledge to brood mass at 16 dph explained ∼12% of variation in brood mass at 16 dph (models 4 and 6; Fig. 1), while model 5, which connected variables serially, only explain 1.4% of variation in brood mass at 16 dph (Table S2).

## DISCUSSION

In this study, parents’ body mass decreased under food restriction without altering furculum fat, but offspring of the food-restricted nests maintained body mass levels similar to control nests and had similar growth rates. These results support our first hypothesis that food-restricted parents are prioritizing more of their limited resources into their reproductive bout and less into self-maintenance. To address potential mechanisms driving fitness outcomes, we asked whether increased glucocorticoid or glucose secretion would account for the observed body mass loss and prioritization toward current reproduction. Food-restricted parents had higher baseline CORT at fledging, but there was no effect of food restriction on baseline glucose, in line with another study on the effects of time-restricted feeding on glucose in zebra finches (Prabhat et al., 2023). Path analyses showed parents’ mass loss from pairing to fledging had a direct, negative effect on baseline CORT at fledging and change in baseline CORT from pairing to fledging. In contrast, change in mass from pairing to fledging had a direct, positive effect on the change in baseline glucose levels from pairing to fledging. Interestingly, blood glucose levels in parents had a negative effect on offspring brood mass at fledging while CORT levels did not. These results suggest that parents that lost more mass during the breeding period also had higher baseline CORT levels, more extreme changes in baseline CORT from pairing to fledging, and less changes in glucose from pairing to fledging. Furthermore, lower changes in glucose were related to heavier broods. Taken together, these results support our second hypothesis that CORT and glucose are potential mechanisms for changes in body mass, but CORT may not be as involved in reproductive effort as glucose. Moreover, food restriction had no effect on the adrenocortical or glucose responses. To our knowledge, this is the first manipulated whole-nest food restriction study showing a relationship between food restriction and outcomes of reproductive trade-offs and providing a mechanism for this relationship in zebra finches.

In our study, food-restricted parents showed higher baseline CORT when the offspring fledged. The fledging period is a particularly demanding time for avian parents, as the offspring are still nutritionally dependent, and their size is comparable to their parents. Thus, the energetic demand on parents to provide sufficient resources to offspring may be magnified and lead to physiological or fitness costs compared to earlier stages of their life history. Indeed, it is likely that food-restricted parents require significantly more energy mobilization to compensate for increased demand with limited resources. As glucocorticoids are shown to facilitate energy mobilization under challenging environments, the observed increase in baseline CORT levels around fledging most likely reflects the need for energy allocation toward provisioning their brood (Bonier et al., 2009) and food searching behavior (Breuner, 1998; Kitaysky et al., 2003; St. Juliana et al., 2017). It would be expected for glucose to follow the same pattern, as an increase in blood glucose is one of the downstream effects of elevated glucocorticoids via reduced glucose reuptake and increased gluconeogenesis (Dimitriadis et al., 1997; Sapolsky et al., 2000; Kuo et al., 2015; Herman et al., 2016). However, our results show that baseline glucose does not follow the same pattern as glucocorticoids and therefore may not be primarily altered by glucocorticoids (Taff et al., 2022; Romero and Beattie, 2022). Alternatively, the effects of glucocorticoids on glucose levels may occur at a different timescale in which our study was not able to detect (Romero and Beattie, 2022). It is true that administration of glucocorticoids to humans and other animals elevates blood glucose levels, but this effect is most likely through intracellular glucocorticoid receptors where the effect on glucose is not seen until 80-100 min (Munck and Koritz, 1962) but could vary across taxa. As stress-induced samples in this study were collected at 29 min, in line with Taff et al. (2022), it is likely that increases in glucose are primarily driven by a positive relationship between catecholamines – another component of the stress response – and thus there may be other sources of energy reallocation (Taborsky and Porte, 1991) and increases in glucose due to CORT likely occur much later than 30 min (Romero and Beattie, 2022). Future studies should consider exploring time courses to describe the relationship between catecholamines, glucocorticoids, and glucose.

In addition to physiological indicators, energy mobilization can also manifest in changes in morphology. The results in this study showed reduced body mass in food-restricted parents but no adverse effects on offspring body mass and growth rate, suggesting that when parents face a sudden food restriction while raising a brood, parents will prioritize their current brood over maintaining their own body mass. This is contrary to another study in zebra finches that suggests parents prioritize self-maintenance when exposed to low quality food (Krause et al., 2017). This is likely due to the differences in how nutritional stress was applied. While Krause et al. (2017) manipulated macronutrient content or food quality, the current study applied a caloric restriction. These contrasts suggest differences in severity of threatened parental survival or differences in age of parents, in which older parents would have lower residual reproductive value compared to younger parents and therefore could alter decisions in life-history trade-offs. In short-lived species, the primary trade-off is between current reproductive bout and survival (Hamel et al., 2010). In our study, we did not measure survival and therefore cannot make conclusions on this trade-off, but we do see that reproductive bout is prioritized over self-maintenance. Further studies are needed to determine whether this short-term trade-off could potentially have long-term consequences such as decreased survival or future reproductive bouts.

The outcome of ecological trade-offs could differ depending on the type of stressor, but more importantly, the timing of exposure. In a study that enlarged brood size and infested nests with ectoparasites at 3 dph, great tits (*Parus major*; another short-lived passerine) favored self-maintenance over their reproductive bout, which is the opposite pattern found in the current study (Wegmann et al., 2015). Alternatively, the outcome could depend on the additive, synergistic, or antagonistic existence of other stressors. Furthermore, the current study is a captive study, in which all other factors of the environment were controlled, while the previous study in great tits was a wild bird study. It is important to note that wild birds may react differently to stressors than captive birds, as there are many other factors in the wild (i.e. variable temperatures, predation, storms) that can contribute to the balance of the trade-off and modifications to the HPA axis to combat environmental challenges. Future studies should further investigate how short-lived species respond to the food restriction during the breeding period and how different types, magnitudes, timing, and durations of stressors can change the outcomes of life history trade-offs.

Interestingly, although food-restricted parents had lower body mass compared to controls, the treatment groups did not differ in furculum fat. These results suggest that the loss in body mass was not due to subcutaneous fat content. Instead, food-restricted parents likely lost their body mass in their gonads. Indeed, it has previously been shown that low nutritional conditions reduce testis size in house finches (*Haemorhous mexicanus*; Valle et al., 2015) and zebra finches (Prabhat et al., 2020). Low nutritional conditions can also reduce reproductive behavior or delay breeding (Wiersma and Verhulst, 2005; Lynn et al., 2010; Prabhat et al., 2023). Taken together, while investing in their current brood, food-restricted zebra finches are likely reducing investment in future reproduction in the short-term by reducing gonad size, thereby decreasing their body mass without reducing furculum fat.

Sexes can differ in the magnitude of their stress response. In this study, we observed sex differences in baseline glucose and the change in glucose between baseline and stress-induced timepoints. Specifically, we saw males had higher baseline glucose and a stronger glucose response than females. This has been observed in mice, although with the opposite pattern (Dockman et al., 2022). It is possible there is a sex-specific glucose response, in which males undergo higher gluconeogenesis than females to use energy more effectively. Further, since males in this study had significantly lower body mass compared to females, this sex difference in baseline glucose and the glucose response may be related to sex differences in body mass, as we see in path analyses that parent body mass loss from pairing to fledging is negatively related to changes in glucose from pairing to fledging. Therefore, males and females may be using energy in different ways during parental care.

Reproductive investment in biparental species under a stressor, such as food restriction, involves energy allocation and time budget of each parent. In this study, with a short-lived species and a food restriction applied postnatally, parents appear to prioritize their current reproductive bout over self-maintenance during the short-term breeding period. There is a lack of studies involving outcomes of reproductive trade-offs in the face of environmental challenges. Many studies measure effects of environmental challenges on reproductive success (i.e. clutch size) or nestling body mass or growth rate, but few measure the same metrics (i.e. body mass) between parents and offspring in parallel and/or determine whether parents truly prioritize their current reproductive bout or self-maintenance during the breeding period. Measuring similar metrics in parents and offspring could show direct comparisons, and thus show clearer prioritization in reproductive trade-offs. Future studies should consider these points to further show relationships between stressors and the outcomes of reproductive trade-offs. The results from this study underscore the importance of energy allocation during breeding when exposed to food restriction and a lack of energy intake from the environment and the importance of parental decision on reproductive outcomes. More studies are warranted to understand the interactions between reproductive investment, parental burden, physiological mediators, and stressors in deciding outcomes of these life history trade-offs.

## MATERIALS AND METHODS

### Animal husbandry

We used a captive colony of zebra finches (*T. castanotis*) housed at Auburn University, Alabama, USA, for this study. This study was approved by Auburn University's Institutional Animal Care and Use Committee (IACUC) (SOP: 2020-3654; 2019-3477; 2022-4062).

### Food restriction treatment

Food restriction treatment was modeled after previous studies (Nowicki et al., 2002; MacDonald et al., 2006; Schmidt et al., 2013; and others) with some modifications. Forty-six adult males and females were paired for a total of 23 nests, in which age (where birds were paired between 7 and 43 months old) and reproductive experience (where birds had 0-3 successful prior breeding attempts) were randomized. Each 16.5″H * 17.5″W * 15″D cage contained a single breeding pair fed *ad libitum* Kaytee Supreme finch seed. Breeding pairs were assigned to either a control (*n*=11) group or a food-restricted (*n*=12) group to maintain randomization of age and reproductive experience. Starting when the average age of the nestlings in a nest was 5 days post-hatch (dph), the control group received *ad libitum* Kaytee Supreme finch seed in a bowl and two tablespoons of egg food (19.0±0.78 g) in a separate dish daily. Food inside of the bowl before blowing husks, after blowing husks, and after filling with more seed was weighed daily, and cages were cleaned of leftover seed daily. Every other day, this leftover seed was also weighed to calculate 24-h consumption. On days uneaten seed outside of the bowl was not weighed, consumption was calculated by averaging the amount consumed the day prior and the day after. The food-restricted nests were given one tablespoon of egg food (9.50±0.39 g) throughout the treatment period and 80% of control-consumed seed for 2 days (from when the average age of the nestlings in the nest was 5 dph to 7 dph), 70% of control-consumed seed for 2 more days (from 7 to 9 dph), and then 60% thereafter. In other words, the food-restricted nests started the full treatment when the average age of the nestlings was 10 dph. To calculate the amount of food for the food restricted nests, we took 60% of the average amount brood-size matched control nests consumed at the same developmental period. For example, the amount of seed consumed by control nests with a brood size of 4 at each developmental age was measured, and food-restricted nests with a brood size of 4 received 60% of this average control consumed amount. This allowed us to account for brood size by design. 60% was chosen after a pilot study where 65% for food-restricted birds had no effect on offspring mass compared to the controls (Coutts and Wada, unpublished observations). A few control nests were set up first to be able to set up these calculations prior to starting food restriction treatment. Thereafter, both control and experimental nests were set up. This treatment continued until the individual nestlings reached 60 dph (after nutritional independence and the beginning of the song crystallization period). After the treatment period ended, all birds received *ad libitum* seed. Nests also received Nystatin (an anti-fungal medication) via their drinking water from the start of treatment until all offspring reached 25 dph as a preventative measure for gastric yeast infection that may occur due to high amounts of stress. During the experiment, 8.5% of control offspring and 8.7% of food-restricted offspring died due to unknown causes. Those that died too early to be sexed were excluded from analyses. Furthermore, one parent that was noted to have blood with high levels of plasma during the fledging blood sample collection and died shortly after was excluded from analyses. As approved by IACUC, animals were monitored for body mass during each timepoint to ensure that they did not sustain body mass loss of 20% from pairing.

### Morphological measurements and blood collection

We measured parents’ body mass, scored furculum fat, and collected blood samples on the day of pairing (prior to the start of any treatment), when their offspring fledged (determined when the youngest nestling reached 18 dph), and when their offspring became nutritionally independent (determined when the oldest nestling reached 44 dph). Body mass and furculum fat scores were measured immediately after completion of blood collection at each time point. Furculum fat was scored on a scale from 1-5 by a single person as a qualitative measurement on the amount of fat observed (similar to techniques in Helms and Drury, 1960; Nolan and Ketterson, 1983; Krementz and Pendleton, 1990). Tarsus length was also measured on the day of pairing as a proxy for body size, in which there was no significant difference between treatment groups (linear mixed models; right tarsus: *F*_1,79_=0.72; *P*=0.398; left tarsus: *F*_1,80_=0.34; *P*=0.563). Thus, body size was considered to be the same across treatment groups. To measure parents’ allocation toward offspring, offspring were measured for body mass at 0, 5 (average age of nestlings, i.e. day of treatment start), 10, 16, 25, 45, and 60 dph. Offspring tarsus length was measured at 5, 10, 16, 25, and 45 dph by taking a picture with a ruler and measuring on ImageJ. This tarsus length measurement was used to calculate growth rate by taking the difference between timepoints and dividing by the number of days between each timepoint.

To quantify baseline and stress-induced levels of corticosterone, we followed a standardized capture/restraint protocol to induce an adrenocortical response (Wingfield et al., 1992; Wingfield, 1994). Using this method, adrenocortical response to a standardized stressor of handling and restraint can be compared between treatment groups and across time (Wada et al., 2008). In short, the initial blood samples were taken from the brachial vein within 3 min of entering the room/disturbance (except for one sample that finished collecting at 3:05). The birds were then placed in an opaque paper bag until 29 min after the initial disturbance, and then a second blood sample was collected that was signified as a stress-induced sample. The average time for the baseline and stress-induced sampling was 2:10±0.0297 and 31:40±0.0012 min (mean±standard deviation) after the initial disturbance. All bleeds started between 7:57 h and 8:35 h. All blood samples were stored at 4°C until the collection tubes were centrifuged at 14,800 ***g*** for 10 min. Separated plasma samples were stored at −80°C until the assay.

### Corticosterone assay

Blood plasma CORT levels were measured using an Enzo Corticosterone ELISA kit (ADI-901-097). This kit has been previously optimized for zebra finches in our lab (Rubin et al., 2021) where 1:40 dilution with 1.5% steroid displacement buffer (SDB) was deemed optimal with 1:20 with 2.5% SDB as a low dilution alternative. Briefly, 14 *μ*L was aliquoted for each sample (1:40 dilution). When sample volume was too low to gather 14 *μ*L, 7 *μ*L was aliquoted instead (1:20 dilution). See Table 1 for the SDB and assay buffer volume adjustments made to the sample depending on how much plasma was available for the assay. Each plate had a standard curve and non-specific binding (NSB) in triplicate, with six wells containing 0% CORT Bound (B0), and samples were run in duplicate for each individual as technical replicates. Every plate was run with the same 800 pg/ml sample standard to investigate inter-plate variation (inter-plate variation=8.4%; intra-plate variation=2.723%). Averages of each duplicate of each individual were used in statistical analyses.

### Blood glucose

We quantified blood glucose levels in baseline and stress-induced samples. Glucose was measured in whole blood using a Reli-On glucose meter (Walmart, Bentonville, AR, USA) that was validated for zebra finches in our lab (Pham et al., in revision). Readings were taken from each individual in duplicate as technical replicates unless there was a lack of blood or the reading was more than 30 mg/dL apart. In the latter case, a third reading was taken unless there was a lack of blood preventing a third reading. If a reading was more than one standard deviation from the mean between the triplicates, that reading was removed, and a new individual mean was used in statistical analyses.

### Statistical analysis

All statistical analyses were performed in R (version 4.3.1, “Beagle Scouts”) using either “nlme” or “ordinal” packages (Pinheiro and Bates, 2000; Christensen, 2023). Statistical significance was determined as *P*<0.05. Assumptions of linearity and homoscedasticity were tested by residual plots, while the assumption of normality was tested by histogram plots of the data for linear models. Normality was further tested by Shapiro–Wilk tests on the residuals of each model. Data in models that violated the assumption of normality were not transformed, as linear models are robust and skewed data still produce unbiased results (Schielzeth et al., 2020). Furthermore, data transformation reduces the interpretability of data (Jacqmin-Gadda et al., 2007; Houle et al., 2011; Grilli and Rampichini, 2015). If models with a sex–treatment interaction had a *P*-value above 0.150, it was removed from the final model. The timepoint–treatment interaction was kept in the model to answer our hypothesis on the effect of food restriction after the start of treatment. In all models, the sex–treatment interaction did not meet the criteria and therefore was removed from the final models. Final models and their variables are described below and listed with their statistical results in Table 2.

To analyze the effect of food restriction on parent body mass, baseline CORT, baseline glucose, absolute change in CORT between baseline and stress-induced (i.e. the adrenocortical response), and absolute change in glucose between baseline and stress-induced (i.e. the glucose response), we used linear mixed models (LMM) with treatment, sex, and timepoint as fixed factors, a timepoint–treatment interaction term, and Bird ID as a random effect to account for repeated measures. To analyze the effect of food restriction on offspring body mass and growth rate, we used a similar LMM with treatment, sex, and timepoint as fixed factors, a timepoint–treatment interaction term, Bird ID nested within Nest ID as a random effect to account for repeated measures and avoid pseudoreplication, and an additional “correlation=corAR1()” argument, an argument that accounts for autocorrelation (when datapoints follow each other, such as an individual's body mass over several ages) due to several timepoints. For the parent body mass and baseline CORT and offspring body mass models, we detected a significant timepoint–treatment interaction, thus, Tukey's post-hoc tests followed using the “emmeans” package (Searle et al., 1980) in R to investigate the effect of treatment at each timepoint. For the other models, the timepoint–treatment interaction term was not significant, so results described are the main effects.

We used a cumulative link mixed model using the “ordinal” package in R, a model that allows regression with non-continuous dependent variables that have an order (Christensen, 2023), such as a fat score, to analyze the effect of food restriction on furculum fat with treatment, sex, and timepoint as fixed factors, a timepoint–treatment interaction term, and Bird ID as a random effect to account for repeated measures. The timepoint–treatment interaction term was not significant, so results described are the main effects.

To test our predictions regarding whether CORT and/or glucose were potential mechanisms for trade-offs between self-maintenance and offspring brood mass, we used structural equation modeling (SEM) using the “sem” package (Fox et al., 2024). We formulated three possible relationships among parents’ mass loss from pair to fledge, CORT, glucose, and brood mass at 16 dph (just before fledging). The first analysis examined relationships with a change in baseline CORT and a change in baseline glucose in parents’ circulation between pairing and when offspring fledged. The same analysis was repeated with baseline CORT and baseline glucose in parents’ circulation when offspring fledged. Models tested are listed in Fig. 1.

## Supplementary Material

10.1242/biolopen.060417_sup1 Supplementary information
