# Supplementary material for: Breeding zebra finches prioritize reproductive bout over self-maintenance under food restriction
Source: Biol Open. 2024 Nov 5;13(11):bio060417. doi: 10.1242/bio.060417 (PMC11556311; doi:10.1242/bio.060417)
Supplement: Supplementary information [file biolopen-13-060417-s1.pdf]

Table S1. Results of structural equation modeling for change CORT and glucose between pair and fledging.

Model	Variation in change in baseline CORT from pair to fledge	Variation in brood mass at 16 dph	Variation in change in baseline glucose from pair to fledge
1	22.0%	29.6%	13.1%
2	13.1%	0.1%	47.4%
3	22.0%	33.3%	47.4%

Table S2. Results of structural equation modeling for baseline CORT and glucose at fledging.

Model	Variation in baseline CORT at fledge	Variation in brood mass at 16 dph	Variation baseline glucose at fledge
4	12.5%	12.3%	0.04%
5	0.04%	1.4%	9.9%
6	12.5%	12.6%	9.9%

Biology Open • Supplementary information
